# Supplementary material for: Genomic dissection of the microevolution of Australian epidemic Bordetella pertussis
Source: Emerg Microbes Infect. 2022 Jun 1;11(1):1460–73. doi: 10.1080/22221751.2022.2077129 (PMC9176669; doi:10.1080/22221751.2022.2077129)
Supplement: Supplemental Material [file TEMI_A_2077129_SM8007.zip › Supplementary_Figure_1.pdf]

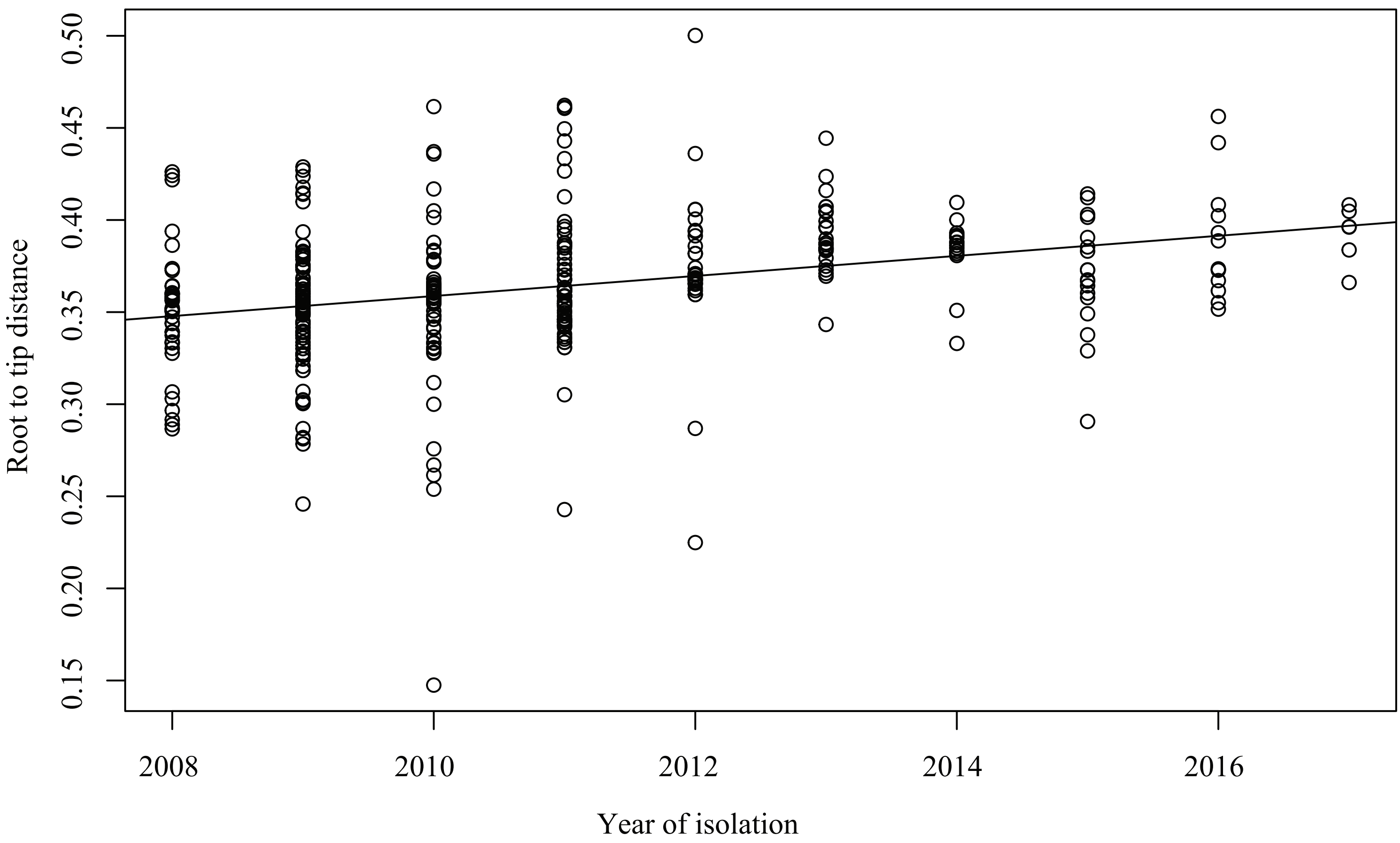

Supplementary Figure 1. Linear regression plot of *B. pertussis* displaying the correlation (R2) between the root-to-tips distance (y-axis) and the date of isolates (x-axis). The root-to-tip distances of individual isolates correlated with their date of isolation. (R2= 0.1048, P < 0.001)
